# Alterations in gray matter volume due to unilateral hearing loss

**DOI:** 10.1038/srep25811

**Published:** 2016-05-13

**Authors:** Xingchao Wang, Pengfei Xu, Peng Li, Zhenmin Wang, Fu Zhao, Zhixian Gao, Lei Xu, Yue-jia Luo, Jin Fan, Pinan Liu

**Affiliations:** 1 Department of Neurosurgery, Beijing Tiantan Hospital, Capital Medical University, No. 6 Tiantan Xili Dongcheng District, Beijing 100050, China; 2 China National Clinical Research Center for Neurological Diseases, No. 6 Tiantan Xili Dongcheng District, Beijing 100050, China; 3 Institute of Affective and Social Neuroscience, College of Psychology and Sociology, Shenzhen University, Nanhai Ave 3688, Shenzhen 518060, Guangdong, P.R. China; 4 Neuroimaging Center, University Medical Center Groningen, University of Groningen, Oude Kijk in ‘t Jatstraat 26, Harmonie Complex, 9712 EK Groningen, The Netherlands; 5 Lab for Neuro-reconstruction, Beijing Neurosurgery Institute, No. 6 Tiantan Xili Dongcheng District, Beijing 100050, P.R. China; 6 Department of Radiation Oncology, Massachusetts General Hospital, Harvard Medical School, Boston, 55 Fruit Street, Boston, Massachusetts, MA 02114, USA; 7 Department of Psychology, Queens College, The City University of New York, 65-30 Kissena Blvd, Flushing, NY, 11367, USA; 8 Department of Psychiatry, Icahn School of Medicine at Mont Sinai, 1 Gustave L. Levy Place New York, NY, 10029, USA; 9 Department of Neuroscience, Icahn School of Medicine at Mont Sinai, 1 Gustave L. Levy Place New York, NY, 10029, USA; 10 Friedman Brain Institute, Icahn School of Medicine at Mont Sinai, 1 Gustave L. Levy Place New York, NY, 10029, USA

## Abstract

Although extensive research on neural plasticity resulting from hearing deprivation has been conducted, the direct influence of compromised audition on the auditory cortex and the potential impact of long durations of incomplete sensory stimulation on the adult cortex are still not fully understood. In this study, using voxel-based morphometry, we evaluated gray matter (GM) volume changes that may be associated with reduced hearing ability and the duration of hearing impairment in 42 unilateral hearing loss (UHL) patients with acoustic neuromas compared to 24 normal controls. We found significant GM volume increases in the somatosensory and motor systems and GM volume decreases in the auditory (i.e., Heschl’s gyrus) and visual systems (i.e., the calcarine cortex) in UHL patients. The GM volume decreases in the primary auditory cortex (i.e., superior temporal gyrus and Heschl’s gyrus) correlated with reduced hearing ability. Meanwhile, the GM volume decreases in structures involving high-level cognitive control functions (i.e., dorsolateral prefrontal cortex and anterior cingulate cortex) correlated positively with hearing loss duration. Our findings demonstrated that the severity and duration of UHL may contribute to the dissociated morphology of auditory and high-level neural structures, providing insight into the brain’s plasticity related to chronic, persistent partial sensory loss.

Sensory perception modulates the adaptation of cortical structures to various sensory experiences^1^,^2^. In previous studies, experience-dependent functional brain plasticity in the primary auditory cortex has been documented in deaf individuals^3^,^4^,^5^, and structural alterations in the deaf have also been found outside the auditory areas^6^,^7^. These findings support the hypothesis that auditory comprehension relies on a distributed network that includes not only the auditory cortex, such as the bilateral superior and middle temporal gyri, but also other “non-auditory” areas, such as the left prefrontal and premotor cortices^8^. In fact, auditory input is critical for cognitive processing to dynamically interact with the environment. A complex combination of perceptual and high-level cognitive factors builds the foundation for integrated auditory processing in individuals with normal hearing^9^,^10^. Accordingly, over time, reduced clarity of peripheral hearing, especially chronic and persistent reductions, may alter the auditory cortex and cause more extensive structural reorganization beyond auditory cortical regions. Although neural plasticity resulting from hearing deprivation has been studied intensively, there is little data on changes in both intra- and extra-auditory cortical structures and their correlations with hearing ability and hearing damage duration in hearing-damaged individuals^11^.

Unlike the congenitally deaf, most individuals with unilateral hearing loss (UHL) suffer from post-lingual hearing damage and have passed the critical period for cortical development. Therefore, any influence on the brain might induce traumatic changes in cortical morphology because of defective compensation. In particular, the ability to capture auditory information is largely preserved in UHL, but changes in auditory perception for further cognitive processing are more complicated^11^,^12^. UHL may affect integral auditory perception and auditory processing for higher-order representations^13^. As a result, various cortices for basic perception and high-level cognition may be subject to a wide range of plastic reorganization. The study of UHL offers a unique opportunity to explore cortical reorganization in response to auditory damage and its consequent relevance to severity and duration. Studying UHL also allows us to examine the direct influence of reduced hearing ability on cortical regions for sensory processing, as well as the consequences of prolonged partial deafness and its potential impact on high-level functions that lead to abnormal communication strategies.

This study used volumetric MRI with voxel-based morphometry (VBM)^14^,^15^,^16^ to examine the volumetric change and reorganization of cortical structures in UHL to gain insight into the impact of UHL severity and duration on neuroanatomical characteristics. In this study, UHL primarily resulted from unilateral acoustic neuroma (AN). Progressive unilateral sensorineural hearing damage is the most frequent initial symptom of AN, and it occurs in more than 90% of AN patients^17^. However, there was no other significant change in the quality of life during the observation period^18^, and preoperative AN offers a unique opportunity to explore the brain plasticity in UHL. We hypothesized that hearing ability and UHL duration would be associated with distinctive degenerations in gross cortical morphology, which may reflect the perceptual input damage and the interaction between perceptual and other cognitive factors.

## Results

### Demographics and measures of auditory characteristics

Table 1 shows the demographics and measures of auditory characteristics of the participants. There were no significant differences in age, gender, education and Mini-Mental State Examination (MMSE) results between the normal controls (NCs) and left and right UHL patient groups (*p* values > 0.06). Most UHL participants had significant hearing damage in the impaired ear (14 left UHL with pure tone audiometry (PTA) ≥ 50 dB and 7 with 50 > PTA ≥ 20 dB; 16 right UHL with PTA ≥ 50 dB and the other 5 with 50 > PTA ≥ 20 dB). UHL patients showed higher PTA values in the impaired ear than the same-side ear in NCs (left UHL: *t* = 6.37, *p* < 0.001; right UHL, *t* = 7.85, *p* < 0.001). However, no significant difference was found between PTA values of unaffected ears in UHL patients and the same-side ears in NCs (*t* = 1.84, *p* = 0.07 for left UHL; *t* = 1.85, *p* = 0.07 for right UHL). There were no significant differences in the duration of hearing damage between left UHL (29.7 ± 10.7 ms) and right UHL (23.4 ± 11.4 ms) (*t* = 0.79, *p* = 0.43). PTA values showed no significant differences between the two patient groups between compromised ears (*t* = 0.23, *p* = 0.82) and contralateral ears with intact hearing (*t* = −0.12, *p* = 0.91). Table 2 shows that UHL participants had significantly higher hearing thresholds for bilateral ears under quiet conditions, and there was no differences between the two UHL groups (*t* = −0.422, *p* = 0.676). There were no significant differences in the signal-to-noise ratio (SNR) between compromised ears of left UHL (30.9 ± 23.2 dB S/N)) and right UHL patients (38.9 ± 24.6 dB S/N)) (*t* = −0.596, *p* = 0.556).

### Volumetric differences between patients and normal controls

**Figure 1:**
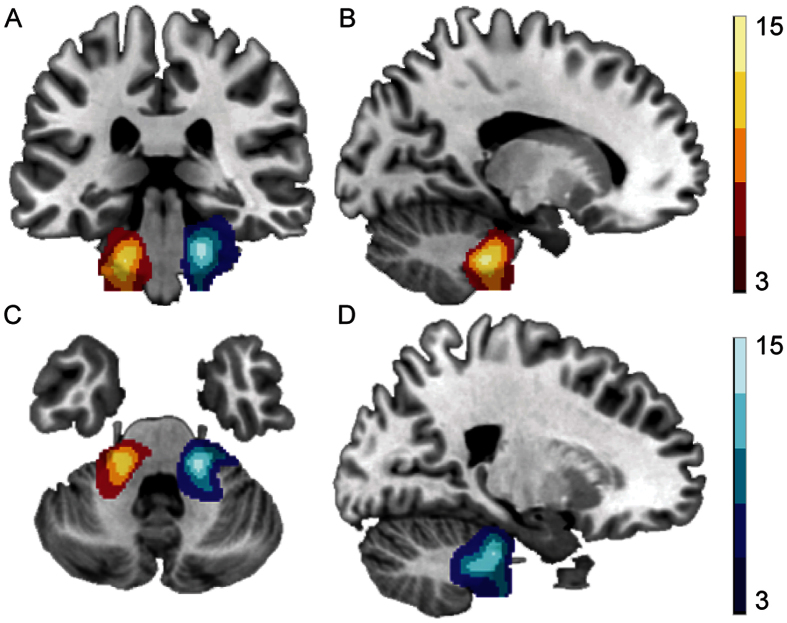
AN tumor volume in left (in red) and right (in blue) UHL patients.

**Figure 2:**
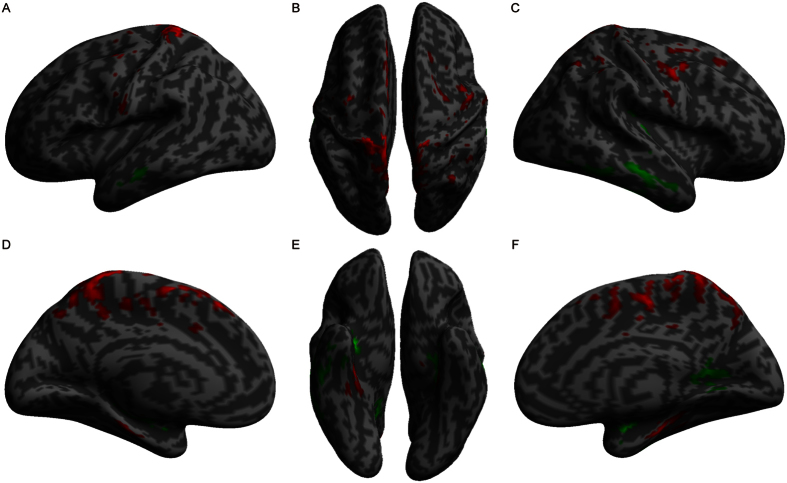
Volumetric differences between patients and normal controls. The red color indicates increased gray matter volume, and the green color indicates decreased gray matter volume in UHL patients compared to normal controls.

Significantly increased AN tumor volume was observed in left and right UHL patients compared to normal controls (Fig. 1). We observed increases in the GM volume of the left supplementary motor area, bilateral precentral gyrus and bilateral postcentral gyrus in UHL patients compared to the cortex of normal controls. In contrast, the GM volume of the bilateral superior temporal gyrus, bilateral middle temporal gyrus, bilateral inferior temporal gyrus, right Heschl’s gyrus, left hippocampus, and right calcarine cortex was decreased (Table 3, Fig. 2). These results indicate that reduced hearing ability in UHL directly influences regions related to auditory processing.

### Volumetric differences between left and right UHL

**Figure 3:**
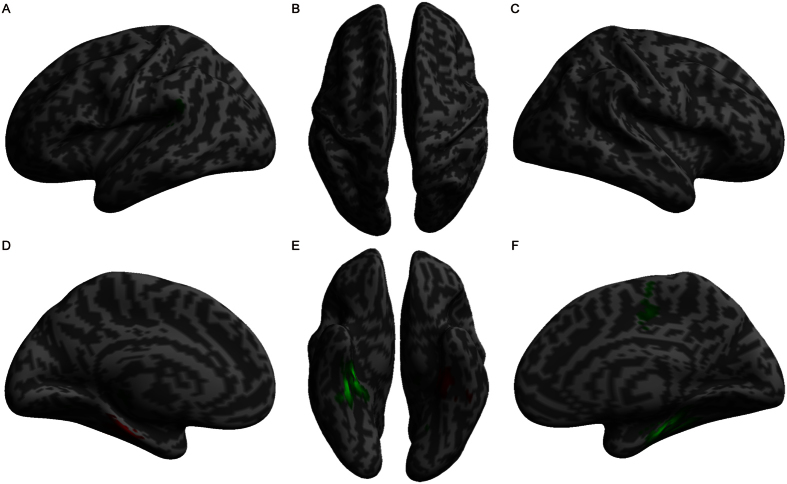
Volumetric differences between left and right UHL. The red color indicates increased gray matter volume, and the green color indicates decreased gray matter volume in left UHL patients compared to right UHL patients.

The GM volume of the right perirhinal cortex in left UHL patients was greater than in right UHL patients (Table 4, Fig. 3). Meanwhile, the GM volume of the left superior temporal gyrus and right supplementary motor area was smaller than in right UHL patients. The most obviously reorganized regions were generally symmetrical, with few differences (i.e., the GM volume in the right supplementary motor area and left superior temporal gyrus in left UHL patients was smaller than in right UHL patients) in GM volumes between left and right UHL. These results demonstrate that UHL is associated with contralateral volume decrease in perirhinal cortex generally; in other words, the GM volume of the perirhinal cortex is specific to the laterality of UHL.

### Correlations between hearing level and gray matter volume

**Figure 4:**
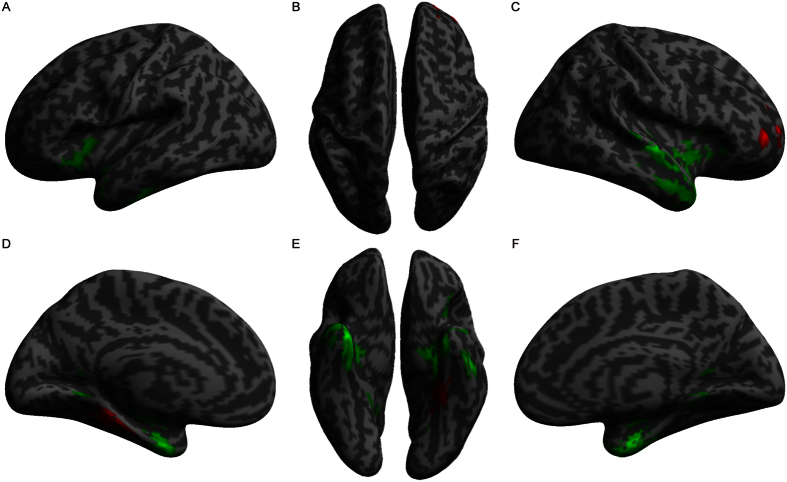
Correlations between hearing level and gray matter volume. The red color indicates voxels with positive relationships, and the green color indicates voxels with negative relationships. The hearing level was calculated as PTA value, and larger values indicate lower hearing ability.

Regression analyses with hearing ability (PTA values, a large value indicates low hearing ability) as the independent variable and GM volume as the dependent variable showed that PTA values (equal to hearing threshold level) were positively correlated with the GM volume of the right superior frontal gyrus and left fusiform gyrus (Table 5, Fig. 4). In contrast, PTA values were negatively correlated with the GM volume of the bilateral superior temporal gyri, left middle temporal gyrus, bilateral inferior temporal gyrus, right Heschl’s gyrus, bilateral anterior insula and bilateral hippocampus. These results demonstrate that greater hearing damage is associated with a more marked reduction of GM in predominantly auditory cortices.

### Correlations between hearing damage duration and gray matter volume

**Figure 5:**
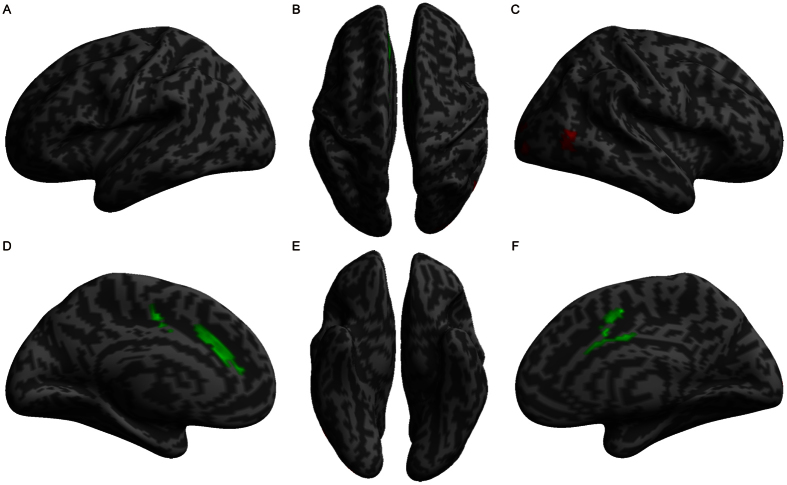
Correlations between hearing damage duration and gray matter volume. The red color indicates voxels with positive relationships, and the green color indicates voxels with negative relationships.

Regression analyses with hearing damage duration as the independent variable and GM volume as the dependent variable revealed that hearing damage duration was positively correlated to the GM volume of the right middle occipital gyrus and right middle temporal gyrus but was negatively correlated to the GM volume of the bilateral anterior cingulate cortex, extending to the supplementary motor area (Table 6, Fig. 5). These data indicate that hearing damage duration is related to the compensation by the visual system and volume reduction in brain regions that subserve high-level cognitive functions.

### Correlations between speech recognition in noise and gray matter volume

**Figure 6:**
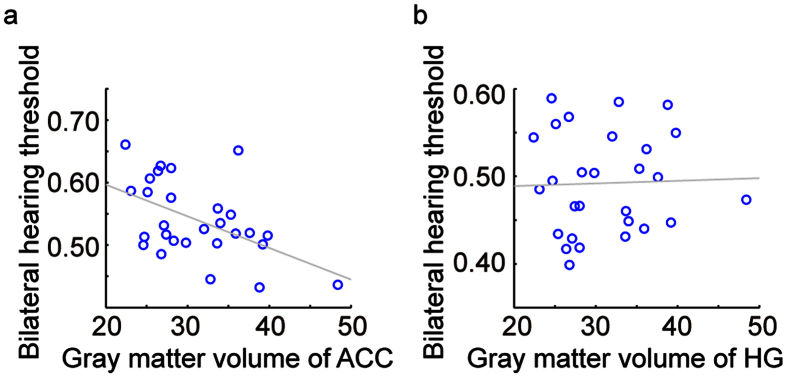
The relationships between the regions of interest (ROIs) and the speech hearing ability. (**a**) Bilateral hearing threshold of speech in quiet was negatively correlated with the GM volume of the anterior cingulate cortex (*r* = −0.51, *p* = 0.005). (**b**) No significant correlation was found between the GM volume of Heschl’s gyrus and the bilateral hearing threshold of speech in quiet (*r* = 0.03, *p* = 0.87). The ROIs were extracted based on the whole-brain analysis of group differences in GM volume between the UHL and NC groups.

For better representation of the types of hearing challenges UHL individuals experience in their daily lives, a speech-recognition in noise test (MHINT) was used to reflect the hearing adaptation besides pure tone. We chose Heschl’s gyrus and the anterior cingulate cortex from differences between UHL and NC groups (shown in Fig. 2) as the regions of interest (ROIs) to examine the relationship between MHINT and GM volume of cognitive and perceptive cortices. The results demonstrate that the bilateral hearing threshold of speech in quiet was negatively correlated with the GM volume of the anterior cingulate cortex (Fig. 6a); however, there was no correlation between speech recognition in noise and Heschl’s gyrus (Fig. 6b). These results confirmed that poorer speech recognition in noise was associated with greater reduction of GM in the anterior cingulate, which is involved in cognitive functions.

## Discussion

In this study, we examined the experience-dependent brain plasticity in UHL participants using an MRI-based morphometric analysis. While the GM volume reductions in primary sensory regions were significantly correlated with subjective hearing ability, the hearing damage duration was related to decreased GM volumes in cortical areas involved in high-order cognitive processing. These results suggest that the severity and duration of UHL respectively contribute to alterations in the GM volume of auditory and high-level functional structures.

Most previous studies have focused on completely deaf individuals, and those volumetric studies revealed either preserved^19^,^20^,^21^ or decreased^7^ GM volume of the auditory cortex in deaf individuals. Our findings with respect to the neuroplasticity of individuals with varying scales of UHL demonstrated the influential role of auditory inputs on GM volume, especially in the temporal lobe. The temporal lobe, which receives and processes auditory information, is the key area for central auditory perception^22^. The correlation between severity of hearing damage and decreased GM volume in temporal lobe in UHL patients suggests that peripheral hearing ability plays a noticeable role in GM volume reduction in the auditory cortex. Similar to the findings that changes in GM density are associated with hearing damage in the elderly^23^,^24^,^25^, cortical structures cannot preserve their typical morphology after unilateral auditory deprivation because of defective compensation for post-lingual unbalanced sensory input, and auditory deprivation gradually attenuates cortical connections in UHL individuals. Consistent with the tonotopic reorganization of the auditory cortex following peripheral hearing loss^26^, our data support the idea that compromised sensory input induces a reorganization of the sensory cortex^27^, especially following asymmetric hearing damage^28^. Furthermore, the brain is organized cooperatively to facilitate responses to sensory input^29^. Cross-modal reorganization in the remaining intact sensory systems likely mediates neural compensation^30^. Increased listening effort is often associated with increased activity in the premotor cortex^31^,^32^,^33^, which is consistent with the increased GM volume in the supplementary motor and precentral gyri in the current study and suggests compensatory neuroplastic reorganization.

Additionally, UHL might disrupt more elaborate auditory information, such as musical perception, vocal communication sounds and spatial and temporal auditory processing. These high-level auditory processes involve many brain regions, particularly the insula, as shown in lesion studies^34^,^35^,^36^,^37^. The insula plays a critical role in multi-sensory integration of emotional factors^38^,^39^,^40^,^41^, and it is a hub that links information from diverse cognitive functional systems^42^. This region plays an important role in several independent but interrelated cognitive control networks^38^,^39^,^43^. In our previous UHL study^13^, we demonstrated increased regional homogeneity in the anterior insular cortex as well as enhanced resting-state functional connectivity between the insula and several key regions of the default-mode network, empathizing the significant reorganization of insular cortex during UHL. Therefore, we infer that the correlation between GM volume and insula of UHL participants originates from the obstruction of the engagement of diverse cognitive processes related to perceptional deficits.

Previous studies have shown that the anatomical and functional changes observed in deaf individuals are present in basic sensory and higher-level cognitive processing^13^,^24^. Likewise, UHL patients suffer from difficult listening conditions in the absence of intact acoustic input, and UHL might induce cognitive dysfunction because unbalanced auditory input affects integral auditory perception^12^ and higher-order representations of auditory processing^2^,^44^,^45^. Thus, neuroplasticity in UHL patients can be mediated by both sensory and cognitive mechanisms, as they cannot communicate through sound as usual and must develop behavioral adaptations. The duration of hearing damage in our study was associated with cortical atrophy mainly in the bilateral anterior cingulate cortex, right superior frontal gyrus and bilateral middle frontal gyrus, which are critical structures for cognitive processing^46^. Consistent with the correlation between hearing damage duration and GM volume, decreased speech recognition ability is associated with a more marked reduction in the GM volume of the bilateral anterior cingulate cortex. The findings that cortices associated with high-level cognition are susceptible to speech-recognition ability support our hypothesis that the prolonged partial hearing damage alters the auditory cortex as well as impact on high-level functions. GM changes in the prefrontal cortex have been associated with impairments in decision-making^47^, executive function^48^,^49^, social cognition^50^, empathizing^51^ and other cognitive control processing^52^ in previous disease-related studies. Accordingly, our findings suggest that abnormal cognitive control may be a traumatic consequence of the reshaping of cortical structures in response to input signals that are aligned with behavioral adaptation^13^,^53^.

None of the post-lingual UHL participants in our study were in the critical period of auditory development; thus, the sensory system may be too stable to adequately modulate plastic modality. Post-lingual UHL induces a decline in the ability to process advanced auditory processing, such as phonological analysis and phonological processing for visual communication (i.e., speech-reading)^54^. This decline may parallel to dynamic cognitive processing, and the higher-order representations would be modified as the duration of hearing damage progresses. Reduced hearing input obstructs the interaction between the brain and the outside world, which desensitizes the detection of the dynamic environment. Thus, we speculate that the perceptional deficit in UHL compels patients to pay more attention to internal information. Therefore, the correlation between frontal lobe GM volume and hearing loss duration in UHL participants may indicate a sensory experience-driven plasticity that resulted at least in part from specific cognitive factors (i.e., down-regulated cognitive processing for perceptual recognition and bottom-up mechanisms developed because of the experienced perceptional deficits).

We also observed an approximate inversion symmetry of cortical reorganization between participants with UHL on different sides. The structural changes in the perirhinal cortex/parahippocampus were specific to the laterality of UHL. The perirhinal cortex is a critical association area for the cross-modal integration of highly processed sensory information^55^. Reduced auditory input impairs sensory integrity and clearly attenuates object-recognition abilities. Therefore, this reduced input enhanced the associative flexibility in the perirhinal cortex to facilitate the identification of environmental stimuli and maintain the balance of auditory information. Eventually, the contralateral neural pathway of auditory processing drives the increased GM volume in anatomically distinguishable substrates, i.e., the perirhinal cortex of the right/left hemisphere that resulted from left/right UHL.

There are some limitations in this study. We investigated the brain morphological changes and their correlations to hearing damage in AN patients. Growth of the tumor in the cerebellopontine angle is likely to change anatomical relationships in the brain. However, the tumors grow subtentorially, thus requiring long-term growth to affect hemispheric structures. Considering patients with UHL without a growing tumor in the brain would increase the probability that the changes in morphometry are not by chance and not due to tumor growth, and further studies including data on UHL in the absence of a neurinoma would be helpful. Moreover, the reorganization of higher-level cognitive processing areas is generally convincing in the face of severe and persistent UHL, and we performed basic speech-in-noise tests to obtain more direct evidence supporting this claim. Future studies may provide detailed information by measuring the higher-order auditory processing more specifically.

In conclusion, our results indicate that UHL induces plasticity in low-level sensory and high-level representation systems to adapt to the impaired auditory input. In addition, brain remodeling occurs in anatomically distinguishable substrates in response to the severity and duration of hearing damage.

## Materials and Methods

### Participants

Patients with unilateral acoustic neuroma (AN) provided consent and were hospitalized in our neurosurgery department (Beijing Tiantan Hospital, Capital Medical University, China) between July 2014 and December 2014. We collected MRI data from 48 patients in total. All the patients were preoperatively diagnosed with UHL resulting from intracranial tumors based on MRI scanning and auditory assessment (pure tone audiometry, described below). Six of the patients were confirmed pathologically as having meningioma and were excluded from the experiment. Therefore, we reported results of the other 42 UHL patients with the same etiology. Twenty-one patients had hearing damage in the left ear, and 21 patients had hearing damage in the right ear. Twenty-four normal control (NC) participants were recruited from the local community in Beijing, with age, gender and education matched to the patients. All the controls also underwent the same MRI and auditory tests. These assessments were necessary for the patients during their clinical management, with no additional cost for them, and the control group received all the tests for free. The study was conducted on the UHL patients with AN preoperatively, and all the UHL participants had undergone the craniotomy to remove the neuromas after the MRI and auditory exam according to the clinical arrangement. All participants were right-handed and reported no previous or current psychiatric disorders (see Table 1 for participant demographics and auditory characteristics). Participants were informed of the requirements and provided written consent prior to participation. The Institutional Review Board of Beijing Tiantan Hospital of Capital Medical University approved this study, and the study procedures were carried out in accordance with the approved programs.

### Clinical assessment

#### Pure tone audiometry (PTA)

We used a standard Hughson-Westlake PTA procedure to identify bilateral hearing threshold levels in each participant. A clinical audiologist collected air-conduction pure-tone audiograms under standard conditions. Audiometric measurements were performed using a GSI-61 audiometer with a TDH39 headphone. The audiological equipment was calibrated. Pure-tone audiometry was conducted at frequencies of 0.25, 0.5, 1, 2, 4 and 8 kHz. Acoustic thresholds of the affected ear were compared to the contralateral unaffected ear at each frequency level. The mean acoustic threshold in speech frequency ([0.5 kHz + 1 kHz + 2 kHz + 4 kHz]/4) was calculated for each participant as the primary audiometric measure. In general, higher PTA values imply worse hearing ability.

#### Mandarin hearing in noise test (MHINT)

MHINT was conducted by using the bilateral implant test system (BLIMP) which was developed by House Ear Institute^56^, University of Hong Kong^57^ and Beijing Tongren Hospital^58^. BLIMP makes the speech and noise signals from the built-in sound card transfer to the external stimulation signal port of a Conera audiometer, and audiometric measurements were performed using a headphone. All the audiological equipment was calibrated using a B & K 4134 pressure-type microphone and a B & K 4153 artificial ear. There are 12 sentence lists in MHINT, each containing 20 sentences, and there are 10 words in each sentence. These sentences were recorded using a male professional voice actor who spoke Mandarin, an unaccented dialect free of noticeable regional influences. At the beginning, the hearing threshold was defined as the participants being able to repeat 50% of the key words in the sentence in quiet. The maximum noise level that cannot impact the 50% accuracy is defined as the background noise, and the starting noise ratio is zero. Then, BLIMP automatically adjusts the speech sound intensity (background noise is fixed, to change the signal-to-noise ratio) according to the participants’ accuracy of repeating the key words in the sentences. The judgment rule was settled as 50–74% in BLIMP (MHINT moves to the next sentence if the accuracy rate is between 50–74%; otherwise, BLIMP turns the speech sound volume up or down). The first four sentences were used as the adaptability test, and speech sounds were adjusted by 4 dB between each pair of sentences. If the first four sentences are all right or all wrong, the test will stop automatically, and MHINT will restart with resetting the intensity of the speech sound. Beginning with the fifth sentence, the adjusted standard is 2 dB, and the left and right ears are tested separately. The final result is the signal-to-noise ratio (SNR, measured as S dB/N), and lower SNRs indicate better speech recognition ability. Unfortunately, because some patients who only speak dialects had difficulty understanding the Mandarin and some patients could not complete the whole test, we only collected MHINT data from 13 left and 15 right UHL patients.

#### Mini-Mental State Examination (MMSE)

An MMSE^59^ was conducted to assess differences in general cognitive ability between the UHL and NC groups. The MMSE is a commonly used clinical screen for cognitive impairment^59^. The MMSE includes 11 items that assess eight categories of cognition: orientation to time, orientation to place, registration, attention and calculation, recall, language, repetition, and complex commands. The maximum score is 30 points, and a low score indicates cognitive impairment.

### MRI acquisition

All structural images were acquired using the same 3.0 Tesla Siemens TRIO scanner (Erlangen, Germany) with a standard head coil in a single scanning session. Head fixation with foam pads and hearing protection with earplugs were used for each participant. T1-weighted sagittal anatomical images were obtained using the following gradient echo sequence: 176 slices, slice thickness/gap = 1/0 mm, inversion time = 900 ms, repetition time = 2300 ms, echo time = 3 ms, flip angle = 7°, number of excitations = 1, field of view = 240 × 240 mm^2^ with an in-plane resolution of 0.9375 × 0.9375 mm^2^.

### VBM analysis

Images were analyzed using the voxel-based morphometry toolbox (VBM8; http://dbm.neuro.uni-jena.de/vbm/) with a statistical parametric mapping package (SPM8; http://www.fil.ion.ucl.ac.uk/spm/software/spm8/). The structural MRI scan of each participant was preprocessed using a modified optimized-VBM-protocol^15^, which may reduce scanner-specific bias^60^. A 12-parameter affine transformation and nonlinear deformations were applied to normalize images to the stereotactic space (152 T1 MNI template, Montreal Neurological Institute). A modulation was applied to the partitioned images by multiplication with the relative voxel volumes or Jacobian determinants of the deformation field to preserve the absolute volume of gray matter within each structure^14^. Modulated images were segmented into gray matter, white matter, and cerebrospinal fluid using a modified mixture model cluster analysis technique^14^. In the current study, we only analyzed GM volume, based on our interests. We applied an isotropic smoothing Gaussian kernel of 8 mm full-width-at-half maximum (FWHM) to the normalized GM segments to reduce signal noise and artifacts from head movements and residual inter-subject variability that was introduced by normalization.

Two-sample *t*-tests were performed to test volumetric differences between left and right UHL patients and between UHL and NC groups. Regression analyses were conducted to examine the relationship between audiometric measures (PTA and MHINT) and GM volume in left and right UHL groups. AFNI AlphaSim (http://afni.nimh.nih.gov/afni/doc/edu) was used for Monte Carlo simulation with 1,000 iterations to correct for multiple comparisons across the thresholded GM mask, which only included voxels with a probability higher than 0.4 in the SPM8 GM template. A cluster extent of 328 contiguous resampled voxels (1.5 × 1.5 × 1.5 mm^3^) was considered significant assuming an individual voxel type I error of p < 0.01, which corresponds to a corrected p < 0.05.

## Additional Information

**How to cite this article**: Wang, X. *et al*. Alterations in gray matter volume due to unilateral hearing loss. *Sci. Rep.* **6**, 25811; doi: 10.1038/srep25811 (2016).

## Figures and Tables

**Table 1:** Demographics and auditory characteristics of different groups.

	**Left UHL**	**Right UHL**	**NC**	***F*** **value**	***p*** **value**
Age (years)	46.4 ± 5.8	44.1 ± 11.2	45.7 ± 4.5	0.380	0.686
Gender	13 f/8 m	11 f/10 m	14 f/10 m	0.399*	0.819
Handedness	R	R	R	NA	NA
Education (years)	12.3 ± 1.7	10.7 ± 2.2	13.6 ± 1.2	3.020	0.056
MMSE	27.0 ± 1.0	27.1 ± 1.3	28.3 ± 0.6	2.014	0.142
Left ear PTA (dB)	67.4 ± 16.7	18.9 ± 2.1	16.1 ± 2.1	1.846^a^	0.072
				−6.365^b^	0.000
Right ear PTA (dB)	18.7 ± 3.1	65.0 ± 13.0	15.2 ± 2.2	1.842^c^	0.072
				7.852^d^	0.000
				−0.120^e^	0.905
Duration of UHL (months)	29.7 ± 10.7	23.4 ± 11.4	NA	0.791**	0.434

Abbreviations: UHL, unilateral hearing loss patients; MMSE, Mini-Mental Status Examination; f, female; m, male; R, right; NC, normal controls; PTA, pure-tone audiometry results of the left/right ear ([0.5 kHz + 1 kHz + 2 kHz + 4 kHz]/4), NA= not available

^*^χ^*2*^ value of Chi-square test.

^**^t value of *t*-test between the duration of left and right UHL.

^a^*t* value of *t*-test between PTA in the left ear of right UHL and NC.

^b^*t* value of *t*-test between PTA in the left ear of left UHL and NC.

^c^*t* value of *t*-test between PTA in the right ear of left UHL and NC.

^d^*t* value of *t*-test between PTA in the right ear of right UHL and NC.

^e^*t* value of *t*-test between PTA in the intact ear of left and right UHL.

**Table 2:** MHINT characteristics of UHL groups.

	**Left UHL**	**Right UHL**	***F*** **value**	***p*** **value**
Bilateral hearing threshold in quiet (dB)	30.9 ± 4.9	31.5 ± 3.0	−0.422	0.676
SNR of impacted ears (dB S/N)	30.9 ± 23.2	38.9 ± 24.6	−0.596	0.556

Abbreviations: UHL, unilateral hearing loss patients; SNR: signal-to-noise ratio.

**Table 3:** Volumetric differences between normal controls and UHL patients.

**Region**	**L/R**	**BA**	**x**	**y**	**z**	**T**	**Z**	***p***	***k***
UHL Patient >NC									
Posterior cingulate cortex	R	31	2	−18	47	6.22	5.47	0.000	15989
Supplementary motor area	L	6	2	11	48	6.02	5.33		
Precuneus	L	5	−3	−48	66	5.97	5.3		
Paracentral lobule	L	4	−8	−35	71	5.67	5.08		
Postcentral gyrus	R	3	20	−39	71	5.55	4.99		
Superior frontal gyrus	L	8	0	27	56	5.23	4.75		
Precentral gyrus	R	6	56	−6	47	4.91	4.5		
Precentral gyrus	L	6	−33	−6	59	4.91	4.5		
Postcentral gyrus	L	4	−30	−27	68	4.76	4.38		
Inferior parietal lobule	R	7/40	45	−45	53	3.91	3.69	0.000	448
UHL Patient <NC									
Hippocampus	R		35	−8	−20	5.34	4.83	0.000	713
Parahippocampal gyrus	R	34	14	−2	−23	2.95	2.84		
Hippocampus	L		−36	−11	−20	4.05	3.8	0.000	533
Superior temporal gyrus	L	22	−50	−12	5	3.21	3.08		
Inferior temporal gyrus	R	20	65	−45	−23	3.64	3.46	0.000	3139
Middle temporal gyrus	R	21	63	−23	−12	3.63	3.45		
Middle temporal gyrus	L	21	−62	−9	−20	3.73	3.53	0.000	789
Inferior temporal gyrus	L	20	−65	−23	−29	2.77	2.68		
Precuneus	R	30	12	−50	9	3.71	3.51	0.000	745
Calcarine cortex	R	19	24	−63	6	2.89	2.79		
Heschl’s gyrus	R		39	−26	14	3.37	3.22	0.001	468
Superior temporal gyrus	R	42	56	−35	14	2.46	2.39		

L, Left; R, right; BA, Brodmann’s area. Height threshold: T = 2.39, *p* < 0.01; extent threshold: *k* = 328.

**Table 4:** Volumetric differences between left and right UHL patients.

**Region**	**L/R**	**BA**	**x**	**y**	**z**	**T**	**Z**	***p***	***k***
Left > Right									
Perirhinal cortex	R	35	−26	−26	−26	6.26	5.50	0.000	2954
Left < Right									
Cerebellum 4 5	L		−9	−44	−9	3.7	3.51	0.000	1051
Cerebellum 6	L		−21	−60	−17	3.2	3.07		
Superior temporal gyrus	L	42	−54	−42	20	3.74	3.54	0.000	731
Supplementary motor area	R	4/6	11	−20	57	3.62	3.44	0.000	488 4

L, Left; R, right; BA, Brodmann’s area. Height threshold: T = 2.39, *p* < 0.01; extent threshold: *k* = 328.

**Table 5:** Correlations between hearing level and gray matter volume.

**Region**	**L/R**	**BA**	**x**	**y**	**z**	**T**	**Z**	**p**	**k**
Positive correlations									
Fusiform gyrus	L	37	−36	−32	−24	5.62	5.05	0.000	2790
Superior frontal gyrus	R	10	33	60	11	4.68	4.32	0.000	981
Negative correlations									
Hippocampus	L		−23	−14	−17	4.65	4.3	0.000	3931
Inferior temporal gyrus	L	20	−53	−18	−35	4.12	3.87		
Superior temporal gyrus	L	22	−48	−3	−11	3.23	3.1		
Middle temporal gyrus	L	20	−45	5	−33	3.20	3.07		
Cerebellum 8	L		−29	−57	−47	5.62	5.05	0.000	3082
Cerebellum 4 5	L		−9	−47	−8	3.84	3.63	0.000	3291
Vermis 6	L		−3	−63	−23	3.62	3.44		
Vermis 3	R		6	−45	−15	3.40	3.25		
Cerebellum 6	L		−21	−69	−26	3.15	3.03		
Precuneus	R	29	11	−50	9	2.68	2.60		
Parahippocampal gyrus	R	34	24	−8	−30	5.05	4.61	0.000	9783
Temporal pole	R	38	27	5	−41	4.56	4.23		
Superior temporal gyrus	R	22	59	−12	−3	4.15	3.89		
Inferior temporal gyrus	R	20	50	8	−39	4.10	3.85		
Hippocampus	R		35	−14	−15	3.49	3.33		
Middle temporal gyrus	R	21	59	−6	−24	3.24	3.1		
Heschl’s gyrus	R		41	−21	12	2.69	2.61		
Insula	R		36	24	11	2.54	2.47		
Insula	L		−35	14	2	3.23	3.1	0.001	970

L, Left; R, right; BA, Brodmann’s area. Height threshold: T = 2.39, *p* < 0.01; extent threshold: *k* = 328.

**Table 6:** Correlations between hearing damage duration and gray matter volume.

**Region**	**L/R**	**BA**	**x**	**y**	**z**	**T**	**Z**	***p***	***k***
Positive correlations									
Middle occipital gyrus	R	19	32	−95	2	3.86	3.54	0.000	403
Middle temporal gyrus	R	19	45	−68	2	3.21	3.01	0.001	358
Negative correlations									
Anterior cingulate cortex	L	24/32	−8	−9	48	4.06	3.69	0.000	2146
Supplementary motor area	R	6	11	−5	48	4.01	3.66		
Anterior cingulate cortex	R	24	2	15	32	3.77	3.47		

L, Left; R, right; BA, Brodmann’s area. Height threshold: T = 2.42, *p* < 0.01; extent threshold: *k* = 328.
